# Diffusion-driven distillation and contrastive learning for class-incremental semantic segmentation of laparoscopic images

**DOI:** 10.1007/s11548-025-03405-1

**Published:** 2025-06-14

**Authors:** Xinkai Zhao, Yuichiro Hayashi, Masahiro Oda, Takayuki Kitasaka, Kensaku Mori

**Affiliations:** 1 Graduate School of Informatics, Nagoya University, Furo-cho, Chikusaku, Nagoya, Aichi Japan; 2 Information Technology Center, Nagoya University, Furo-cho, Chikusaku, Nagoya, Aichi Japan; 3 School of Information Science, Aichi Institute of Technology, 1247 Yachigusa, Yagasa-cho, Toyota, Aichi Japan; 4 Research Center for Medical Bigdata, National Institute of Informatics, 2-1-2 Hitotsubashi, Chiyoda-ku, Tokyo Japan

**Keywords:** Class-incremental semantic segmentation, Diffusion model, Laparoscopic image, Contrastive learning

## Abstract

**Purpose:**

Understanding anatomical structures in laparoscopic images is crucial for various types of laparoscopic surgery. However, creating specialized datasets for each type is both inefficient and challenging. This highlights the clinical significance of exploring class-incremental semantic segmentation (CISS) for laparoscopic images. Although CISS has been widely studied in diverse image datasets, in clinical settings, incremental data typically consists of new patient images rather than reusing previous images, necessitating a novel algorithm.

**Methods:**

We introduce a distillation approach driven by a diffusion model for CISS of laparoscopic images. Specifically, an unconditional diffusion model is trained to generate synthetic laparoscopic images, which are then incorporated into subsequent training steps. A distillation network is employed to extract and transfer knowledge from networks trained in earlier steps. Additionally, to address the challenge posed by the limited semantic information available in individual laparoscopic images, we employ cross-image contrastive learning, enhancing the model’s ability to distinguish subtle variations across images.

**Results:**

Our method was trained and evaluated on all 11 anatomical structures from the Dresden Surgical Anatomy Dataset, which presents significant challenges due to its dispersed annotations. Extensive experiments demonstrate that our approach outperforms other methods, especially in difficult categories such as the ureter and vesicular glands, where it surpasses even supervised offline learning.

**Conclusion:**

This study is the first to address class-incremental semantic segmentation for laparoscopic images, significantly improving the adaptability of segmentation models to new anatomical classes in surgical procedures.

**Supplementary Information:**

The online version contains supplementary material available at 10.1007/s11548-025-03405-1.

## Introduction

**Fig. 1:**
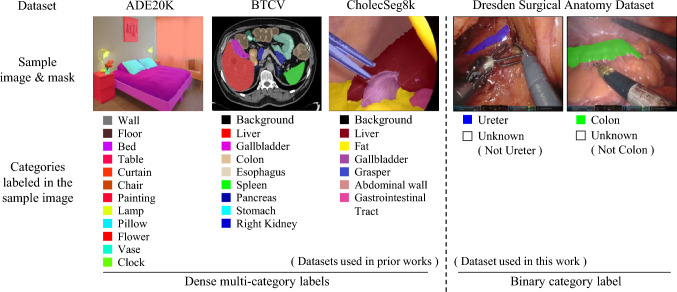
Dataset comparison. Unlike the ADE20K [20], BTCV [21], and CholecSeg8k [3] datasets, where new and old categories are annotated within the same images, the Dresden Surgical Anatomy Dataset [22] introduces new categories on separate images with binary segmentation labels, making it better aligns with the practical clinical setting for CISS in laparoscopic images

Laparoscopic surgery, recognized for its minimally invasive approach, plays a pivotal role in modern treatment of a wide range of diseases, including cholecystectomy, gastrectomy, and intestinal resection [1]. The development of deep learning technologies has remarkably advanced the automatic analysis of abdominal anatomical structures, potentially elevating the quality and safety of surgical interventions. Notably, datasets such as HeiChole [2] and CholecSeg8k [3] have facilitated the researches [4, 5] about laparoscopic anatomy segmentation. However, differing surgical procedures involve distinct categories of anatomical structures. For instance, gastrectomy primarily involves the stomach, and accurately localizing neighboring organs such as the small intestine and spleen is essential for guiding the surgical approach [6]. Similarly, intestinal resection focuses on intestinal veins, the inferior mesenteric artery, and preventing ureter injuries [7]. Consequently, tailored image segmentation models for specific anatomical structures is crucial for diverse surgical procedures and patient conditions. Class-incremental semantic segmentation (CISS) offers a promising solution by enabling efficient and adaptive segmentation. CISS allows the model to continually learn and adapt to new classes of anatomical structures without forgetting previously learned information, making it ideal for the dynamic environment of surgical applications.

In the related works, many CISS methods [8–19] have been developed for both natural [20] and medical [3, 21] image datasets. However, as Fig. 1 illustrates, existing research typically adds new class annotations to images from previous training sets. In clinical setting, incremental learning typically involves providing new data and corresponding annotations separately, rather than retaining and reusing patient images to add new annotations, because the latter approach poses risks of unauthorized access and confidentiality breaches. Similarly, a recently released dataset, the Dresden Surgical Anatomy Dataset [22], introduces new categories on distinct images through binary category label, better aligning with clinical application requirements. Besides, laparoscopic images also pose challenges with their narrow field of view, poor foreground–background contrast, and class imbalance [23, 24]. These factors present significant challenges for training even offline segmentation model [25], and for incremental learning, they reduce the effectiveness of techniques like pseudo-labeling [8] and background-based methods [9], which rely on clearer distinctions and more consistent visual information across images. In addition, privacy concerns with medical images prevent the use of sample replay methods [10, 11]. To address these issues, we use generative replay [12], which creates synthetic images to mimic real data. This method avoids privacy risks and improves model training with diverse, high-quality examples.

Recently, diffusion models [26] have achieved remarkable success in generating high-quality images, and some of the latest work [19] exploring their application in continual learning. Inspired by these studies, we use a diffusion model to generate a large volume of high-quality laparoscopic images, combining knowledge distillation and contrastive learning for class-incremental learning aimed at segmenting laparoscopic anatomical structures. Specifically, our methodology begins by training a segmentation model and a diffusion-based image generation network with laparoscopic images. During continual learning, the diffusion model serves to generate a diversified dataset for subsequent learning phases. We incorporate a knowledge distillation strategy to preserve the memory of previously learned categories and employ contrastive learning between generated and real images to further refine the model.

**Fig. 2:**
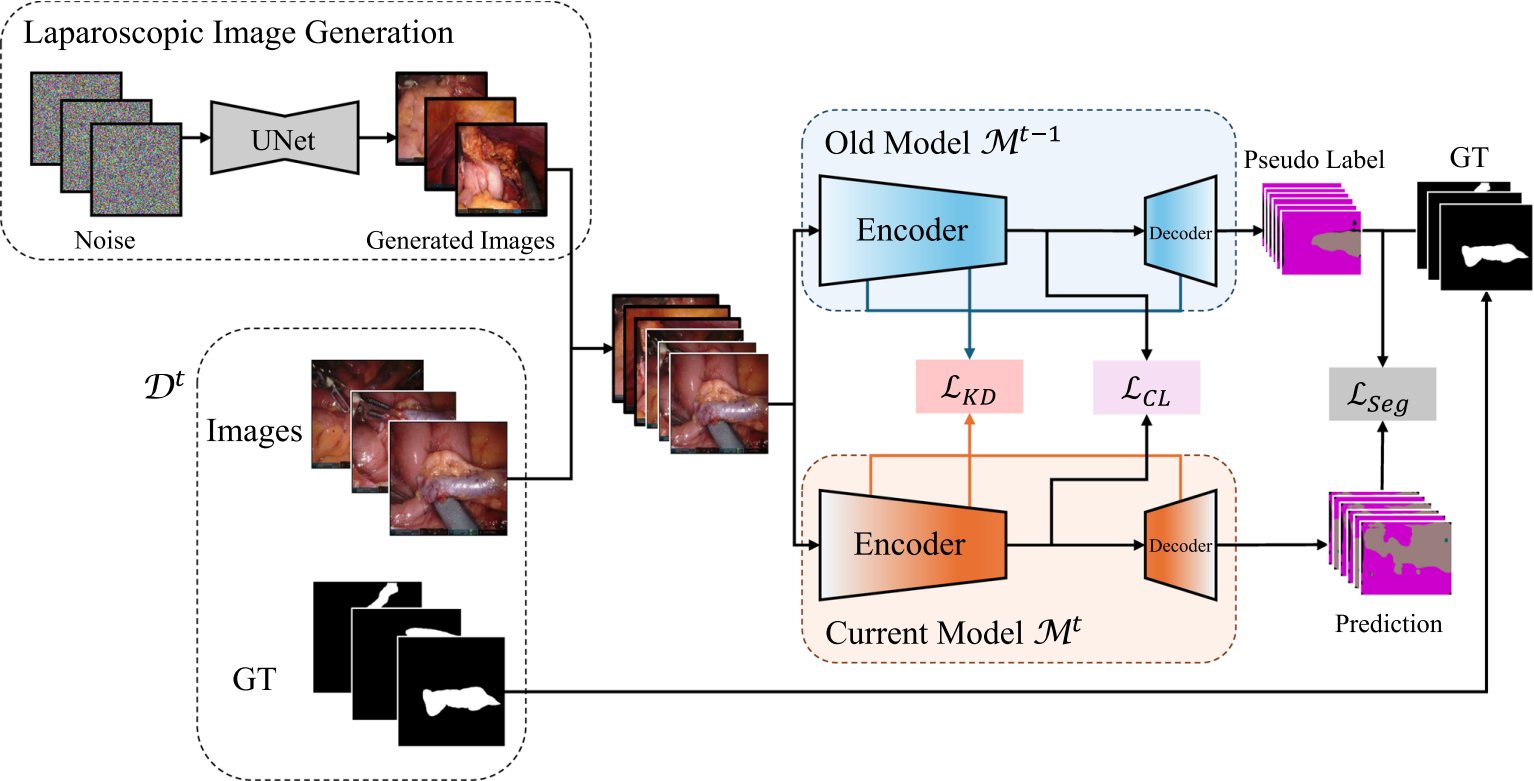
Overview of proposed method. In our approach, we leverage a diffusion model to generate realistic laparoscopic images. Additionally, we employ a framework that combines distillation learning and contrastive learning. This approach optimizes the relationship between real and generated images, effectively balancing the retention of existing knowledge with the acquisition of new insights

In summary, our main contributions are summarized as follows: (1) We creatively address a class-incremental laparoscopic anatomical structure segmentation task which presents significant challenges due to the use of binary labeling. (2) To address the challenges posed by binary category annotation and variations in appearance, we propose a diffusion-driven framework comprising with 3 components for effective incremental learning. (3) Through extensive experiments on a public dataset, we demonstrate that directly applying existing state-of-the-art (SOTA) methods to laparoscopic images results in significant performance degradation, whereas our method significantly improves performance, outperforming other existing approaches.

## Method

This paper addresses class-incremental semantic segmentation of laparoscopic images, aiming to train a segmentation model to recognize new anatomical classes without forgetting prior ones, independent of the initial dataset. Our approach structures the training into $T+1$ steps, starting from an initial step followed by $T$ incremental step. In each step $t$, the model, denoted by $\mathcal {M}^t$, is trained on a dataset $\mathcal {D}^t$ with $\textbf{x}^t$ as the input image and $\textbf{y}^t$ as its corresponding ground-truth segmentation map. Moreover, to prevent catastrophic forgetting, we utilize a diffusion model to generate a set of synthetic images, denoted by $\{\textbf{x}'_0\}_{i=1}^N$, where *N* represents the total number of synthetic images generated. For training at step $t$ ($t>0$), the training set is the concatenation of two image sets: the new generated images $\{\textbf{x}'_0\}_{i=1}^N$ and the current step’s images $\mathcal {D}^t$, thus enhancing the model’s ability to learn new and retain old classes. An overview of each incremental step is shown in Fig. 2.

### Unconditional laparoscopic image generation

In our approach, the primary task is to synthesize additional laparoscopic images $\{\textbf{x}^{\prime }_0\}$ to diversify our training dataset for incremental learning. We utilize the denoising diffusion probabilistic model (DDPM) [26], a generative model that synthesizes realistic images by gradually denoising. Specifically, the objective of the DDPM training phase is to develop an accurate model of the noise characteristics embedded in the data. This learning is facilitated by an iterative process that begins by sampling a real laparoscopic image $\textbf{x}_0$. During training, a diffusion step $s \in \{1, \ldots , S\}$ is selected, and noise $\varvec{\epsilon } \sim \mathcal {N}(\textbf{0}, \textbf{I})$ is added. The model parameters are refined by calculating the discrepancy between the predicted noise and the injected noise:

1 $$\begin{aligned} \nabla _\theta \left\| \varvec{\epsilon } - \varvec{\epsilon }_\theta \left( \sqrt{\bar{\alpha }_s} \textbf{x}_0 + \sqrt{1 - \bar{\alpha }_s} \varvec{\epsilon }, s\right) \right\| ^2, \end{aligned}$$

where $\alpha _s = 1 - \beta _s$ indicating the proportion of the original signal retained at step $s$, $\beta _s$ is a variance schedule for each diffusion step, and $\bar{\alpha }_s$ is the cumulative product of $\alpha _s$ from step 1 to $s$. The neural network $\varvec{\epsilon }_\theta$, parameterized by $\theta$, is trained to predict the noise added at each step, crucial for accurately reversing noise during sampling.

The sampling phase aims to synthesize realistic laparoscopic images by reversing the learned noise distributions. Starting with a noise image $\textbf{x}^{\prime }_S \sim \mathcal {N}(\textbf{0}, \textbf{I})$, the model reconstructs cleaner images in reverse:

2 $$\begin{aligned} \textbf{x}^{\prime }_{s-1} = \frac{1}{\sqrt{\alpha _s}} \left( \textbf{x}^{\prime }_s - \frac{1-\alpha _s}{\sqrt{1-\bar{\alpha }_s}} \varvec{\epsilon }_\theta (\textbf{x}^{\prime }_s, s) \right) + \sigma _s \textbf{z}, \end{aligned}$$

where $\textbf{z} \sim \mathcal {N}(\textbf{0}, \textbf{I})$ if $s > 1$, otherwise $\textbf{z} = \textbf{0}$, and $\sigma _s$ represents the standard deviation determined by the noise schedule. This reverse diffusion process continues until $\textbf{x}^{\prime }_0$, the final synthetic laparoscopic image, is synthesized, thereby enriching the dataset to improve the robustness and generalization of the model in class-incremental learning scenarios, particularly by increasing data diversity, balancing class representation, and enhancing training effectiveness.

### Knowledge distillation for laparoscopic images

To enhance class-incremental learning for laparoscopic image segmentation, we employ dense alignment distillation on all aspects (DADA) method from IDEC [17] as the distillation network backbone. This method efficiently distills knowledge across both intermediate layers and output logits, ensuring accurate pixel classification. The inputs are processed concurrently by the static previous model, $\mathcal {M}^{t-1}$, and the trainable current model, $\mathcal {M}^t$, with atrous spatial pyramid pooling generating context-rich embeddings for effective feature distillation.

The DADA method evaluates similarities between the intermediate features embedding $\{\textbf{e}^{t-1}_l\}_{l \in L}$ from $\mathcal {M}^{t-1}$ and $\{\textbf{e}^{t}_l\}_{l \in L}$ from $\mathcal {M}^t$, aiding $\mathcal {M}^t$ in inheriting and refining its predecessor’s features. Knowledge distillation is implemented using a weighted loss across selected layers and output logits:

3 $$\begin{aligned} \mathcal {L}_{KD} = \sum _{l \in L} \varvec{\omega }_l \cdot d(\textbf{e}_l^{t-1}, \textbf{e}_l^t) \end{aligned}$$

where $L$ represents the intermediate and output layers of network $M$ involved in distillation, $\varvec{\omega }_l$ the layer weights, and $d(\cdot ,\cdot )$ the Euclidean distance.

### Contrastive feature discrimination

To enhance the model’s ability to distinguish features based on their relevance to specific classes and their source (real or synthetic), we employ an image-level contrastive learning strategy. This strategy utilizes encoded features from both current and previous models. Specifically, for any laparoscopic image $\textbf{x}$, we obtain its encoded features $\textbf{z}^{t} = \mathcal {M}^t(\textbf{x})$ from the current model $\mathcal {M}^t$, and $\textbf{z}^{t-1} = \mathcal {M}^{t-1}(\textbf{x})$ from the previous model $\mathcal {M}^{t-1}$. Similarly, for a synthetically generated image $\textbf{x}^{\prime }$, its features are $\textbf{z}^{\prime ^{t}} = \mathcal {M}^t(\textbf{x}^{\prime })$ and $\textbf{z}^{\prime ^{t-1}} = \mathcal {M}^{t-1}(\textbf{x}^{\prime })$.

These features are projected into a new feature space using a projection head $p(\cdot )$, which facilitates effective feature discrimination. In this space, positive pairs consist of features extracted from the same input image by both models $M^{t-1}$ and $M^t$. Negative pairs are constructed by pairing features from a real image processed by current model with features from a synthetic image processed by the current or the previous model.

The contrastive loss for a single positive pair $\left( \textbf{z}^{t}, \textbf{z}^{t-1}\right)$ is defined as follows:

4 $$\begin{aligned}&\mathcal {L}_{CL}\left( \textbf{z}^{t}, \textbf{z}^{t-1}\right) \nonumber \\&\ = -\log \frac{\exp \left( p(\textbf{z}^{t}) \cdot p(\textbf{z}^{t-1}) / \tau \right) }{\exp \left( p(\textbf{z}^{t}) {\cdot } p(\textbf{z}^{t-1}) / \tau \right) {+} \sum _{\textbf{z}^{\prime } \in \{\textbf{z}^{\prime ^{t}}, \textbf{z}^{\prime ^{t-1}}\}} \exp \left( p(\textbf{z}^{t}) {\cdot } p(\textbf{z}^{\prime }) / \tau \right) } \end{aligned}$$

where $\tau$ is a temperature scaling parameter that adjusts the sharpness of the distribution, facilitating the differentiation between similar and dissimilar feature pairs. This approach ensures that features from the same image are more similar to each other than to features from different images, thus enhancing the model’s discriminative capabilities in identifying relevant features from laparoscopic images.

**Table 1:** Comparison of Dice scores by class and step follow the setups in Sect. 3.1.2

Method	7–2 (2 steps)			7–2–2 (3 steps)				7–4 (2 steps)		
	1–7	8–9	1–9	1–7	8–9	10–11	1–11	1–7	8–11	1–11
DeepLabV3+ Framework										
*Fine tuning*	0.00	16.10	3.58	0.00	0.00	21.35	3.88	0.00	20.55	7.47
	±0.00	±7.67	±7.61	±0.00	±0.00	±3.61	±8.38	±0.00	±7.35	±10.83
MiB [9]	45.73	17.39	39.43	37.36	18.33	32.17	32.96	46.25	27.12	39.29
	±10.82	±7.92	±15.61	±13.74	±6.92	±8.32	±13.89	±11.06	±12.61	±14.85
PLOP [13]	40.68	16.48	35.30	31.23	13.72	25.92	27.08	40.33	24.42	34.54
	±13.84	±7.55	±16.21	±14.02	±8.01	±9.27	±14.00	±14.00	±11.56	±15.23
SSUL [15]	55.16	34.42	50.55	48.30	27.75	41.15	43.26	55.13	39.07	49.29
	±23.12	±11.80	±22.82	±21.81	±12.43	±2.40	±19.81	±23.92	±8.59	±21.14
InSeg [14]	57.84	33.95	52.53	52.25	27.33	40.93	45.66	53.40	37.76	47.71
	±23.22	±9.15	±23.17	±22.55	±10.47	±1.90	±20.9	±20.45	±10.38	±19.02
NeST [16]	57.76	28.89	51.34	56.56	29.11	37.20	48.05	58.72	28.59	47.77
	±18.49	±7.52	±20.56	±19.69	±7.80	±6.67	±19.96	±11.20	±5.12	±17.31
IDEC [17]	63.80	32.53	56.85	51.05	25.17	44.81	45.21	64.86	40.59	56.03
	±16.34	±16.10	±20.84	±21.36	±15.35	±3.31	±20.74	±16.69	±11.31	±18.98
**Ours**	**68**.**86**	33.70	**61**.**05**	**65**.**26**	29.08	45.68	**55**.**12**	**68**.**79**	**47**.**34**	**60**.**99**
	±16.20	±8.58	±20.83	±18.91	±9.00	±1.60	±21.15	±16.73	±9.92	±17.90
*Offline*	69.83	28.76	60.70	69.24	28.95	58.25	59.92	69.24	43.60	59.92
	±9.26	±12.60	±19.84	±9.95	±10.15	±2.65	±17.70	±9.95	±16.42	±17.70
ViT Encoder + Mask Decoder (MedSAM) Framework										
MBS [18]	62.45	**47**.**06**	56.59	60.66	19.90	**48**.**20**	50.98	61.57	39.69	53.61
	±28.18	±10.98	±27.65	±27.75	±2.79	±2.23	±27.00	±19.60	±15.98	±21.17
**Ours**	64.29	43.87	59.75	62.88	**33**.**11**	47.37	54.65	64.34	40.74	55.76
	±14.43	±9.87	±19.05	±14.29	±12.61	±1.35	±17.21	±20.59	±10.56	±20.96
*Offline*	64.86	28.35	56.74	64.75	31.74	48.16	55.73	64.75	39.95	55.73
	±12.53	±10.11	±19.37	±13.23	±10.58	±1.39	±17.29	±13.23	±11.15	±17.29

*Offline* methods are trained with all data available at once without incremental learning steps. Highest results and second highest results are highlighted in bold and underlined, respectively

### Overall loss function

The integrated loss function, $\mathcal {L}_{\text {overall}}$, supports CISS by combining three losses:

5 $$\begin{aligned} \mathcal {L}_{\text {overall}} = \mathcal {L}_{\text {Seg}} + \mathcal {L}_{\text {KD}} + \mathcal {L}_{\text {CL}}, \end{aligned}$$

where $\mathcal {L}_{\text {Seg}}$ represents the weighted cross-entropy loss, adjusted for class frequency to tackle class imbalance, as in previous work [25]. In the initial training phase, only $\mathcal {L}_{\text {Seg}}$ is applied. During incremental learning steps, $\mathcal {L}_{\text {Seg}}$ remains actively employed and is augmented by the integration of pseudo-labels from the previous model with current ground-truth annotations. This integration, applied to both generated and real images, leverages high-confidence data to significantly enhance the model’s segmentation accuracy. $\mathcal {L}_{\text {KD}}$ measures the knowledge distilled from the previous to the current model, ensuring retention of learned features. Meanwhile, $\mathcal {L}_{\text {CL}}$ enhances the model’s capability to distinguish between diverse feature representations across both new and previously learned classes.

## Experiments

We assess our CISS framework on public laparoscopic datasets, comparing it with leading methods from other fields to demonstrate our method’s efficacy. Extensive ablation studies further validate our core components.

### Datasets and implementation details

#### Dataset overview

All of our experiments are conducted on the Dresden Surgical Anatomy Dataset (DSAD) [22], which includes 13,195 meticulously annotated laparoscopic images across 11 distinct abdominal anatomical structures from 32 laparoscopic surgeries. This dataset, featuring an almost equal allocation of approximately 1000 images per category, provides pixel-wise annotations for the most prominent anatomical structure in each image, while other structures remain unlabeled. This annotation strategy not only supports the practical application of CISS in real-world clinical settings, but also mirrors the challenges encountered in actual surgical environments. We follow the officially recommended training-validation-test splits [22, 27]: 21 cases for training, 3 for validation, and 8 for testing. Details are in the supplementary material.

**Table 2:** Comparison of Dice scores for all categories after incremental learning of experiment 7–4 (2 steps) using the DeepLabV3+ framework

	Step 0							Step 1				Mean
	AWL	COL	LIV	PAN	SIN	SPL	STO	URE	VGL	IMA	INV	
DeepLabV3+ Framework												
*Fine tuning*	0.00	0.00	0.00	0.00	0.00	0.00	0.00	9.01	23.54	29.14	20.51	7.47
MiB [9]	68.60	40.68	52.86	33.69	43.61	35.67	48.62	10.18	25.76	26.79	45.76	39.29
PLOP [13]	68.46	37.28	45.73	21.95	43.61	26.51	38.75	8.98	23.05	24.05	41.58	34.54
SSUL [15]	79.98	58.26	71.49	40.84	74.76	52.47	4.99	24.62	40.95	44.43	46.43	49.02
InSeg [14]	78.42	63.07	47.10	44.63	72.66	55.89	12.01	20.36	39.64	44.07	46.98	47.71
NeST [16]	75.68	50.75	73.85	**45.61**	60.85	47.93	56.38	21.70	33.77	25.63	33.27	47.77
IDEC [17]	83.14	67.14	76.50	29.27	77.59	58.88	61.49	21.61	46.65	51.01	43.10	56.03
**Ours**	**86.87**	**74.54**	74.93	31.82	**80.49**	**71.33**	**61.59**	30.85	**48.80**	53.05	**56.66**	**60.99**
*Offline*	78.40	73.00	70.60	46.30	77.00	71.50	67.90	18.80	39.10	55.60	60.90	59.92
ViT Encoder + Mask Decoder (MedSAM) Framework												
MBS [18]	76.44	74.24	**82.24**	37.01	79.12	48.76	33.20	**32.21**	17.10	**53.36**	56.07	53.61
**Ours**	82.34	72.32	78.27	43.29	80.15	70.37	23.64	23.15	42.51	46.61	50.70	55.76
*Offline*	72.62	73.97	80.08	48.53	76.54	45.82	55.66	21.16	42.32	46.77	49.55	55.73

Highest results and second highest results are highlighted in bold and underlined, respectively

**Table 3:** Ablation studies of knowledge distillation (KD), data generation (DG), and contrastive learning (CL) under DeepLabV3+ and MedSAM frameworks

Method	DeepLabV3+ Framework			MedSAM Framework		
	1–7	8–11	1–11	1–7	8–11	1–11
KD	62.33	45.16	56.09	61.86	35.93	52.43
KD+DG	66.94	42.80	58.16	63.25	36.66	53.58
KD+DG+CL	68.79	47.34	60.99	64.07	40.30	55.43

#### CISS experimental setup

Our CISS experiments on DSAD focused on segmenting 1. abdominal wall (AWL), 8 abdominal organs (2. colon (COL), 3. liver (LIV), 4. pancreas (PAN), 5. small intestine (SIN), 6. spleen (SPL), 7. stomach (STO), 8. ureter (URE), 9. vesicular glands (VGL)), and 2 vessel structures (10. inferior mesenteric artery (IMA), 11. intestinal veins (INV)). We tested three incremental learning scenarios: A 2-step with a 7–2 split (classes 1–7 in initial step 0, classes 8–9 in incremental step 1), a 3-step with a 7–2–2 split (classes 1–7 in initial step 0, classes 8–9 in incremental step 1, and classes 10–11 in incremental step 2) and a 2-step with a 7–4 split (classes 1–7 in initial step 0, classes 8–11 in incremental step 1).

#### Implementation details

For our experiments, images were resized to $256 \times 256$ pixels. Laparoscopic images were synthesized using the unconditional diffusion model, DDPM [26]. The training set was identical to that used for training the step 0 segmentation network. The image generation network was optimized using the AdamW optimizer with a initial learning rate of 1e−4 for 150 epochs. We used $N=1000$ generated images, each produced using $S=1000$ sampling steps. The segmentation network was evaluated using two frameworks: a ResNet-101 backbone within the DeepLab-V3+ [28] framework, and a framework employing a ViT [29] encoder and mask transformer decoder, same as MedSAM [30]. The DeepLab-V3+ model was optimized using stochastic gradient descent (SGD) with a starting learning rate of 1e−2, while the ViT-based model used a learning rate of 1e−3. Both models were trained for 50 epochs for each incremental learning step, with a batch size of 16. Both networks were implemented in PyTorch and run on two NVIDIA RTX A6000 Ada GPUs. For fair comparison, the same backbone model, supervised training loss, dataset division and epochs are used for all methods. The code is available at github.com/MoriLabNU/DDDC-CL.

**Fig. 3:**
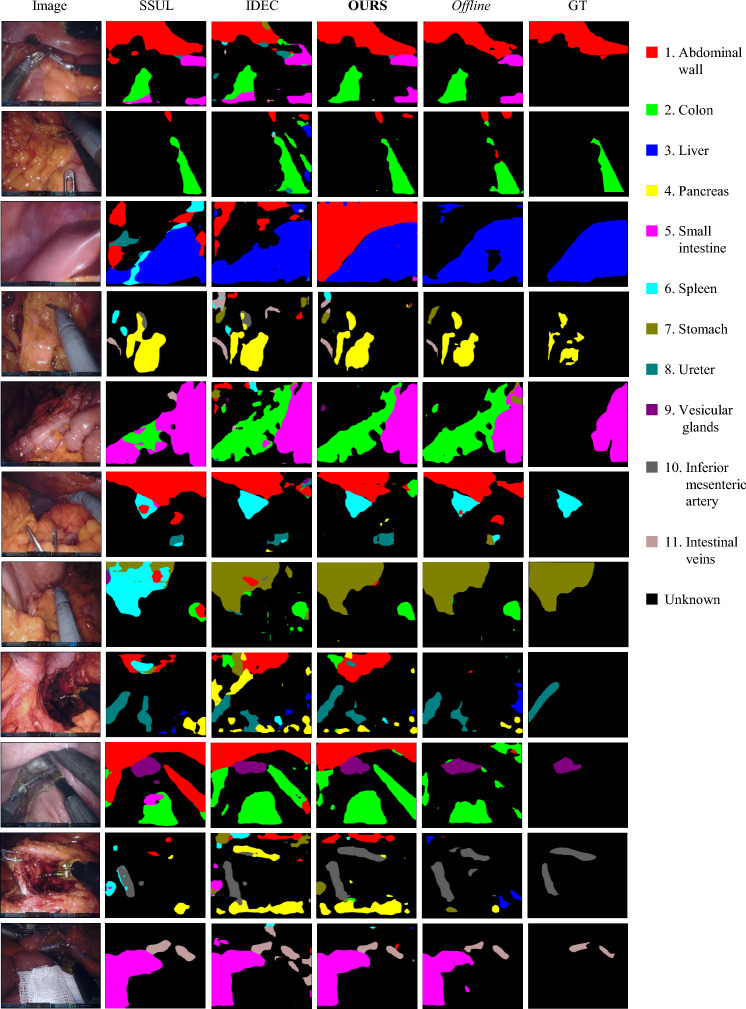
Experimental results of our method and others. Given the dataset provides ground truth for only one type of object, assessing the segmentation accuracy for other regions is problematic. Consequently, we include predictions from a fully supervised, offline approach as a benchmark for comparison

### Quantitative comparison

Following the setups in Sect. 3.1.2, we adapted existing SOTA CISS methods to laparoscopic image segmentation and compared them with our approach. DeepLabV3+ framework-based methods including *fine tuning*, MiB [9], PLOP [13], SSUL [15], InSeg [14], IDEC [17], NeST [16], and MedSAM framework-based method MBS [18] were incorporated. Dice scores from three experimental setups, detailed in Table 1, demonstrate our method’s consistent superiority over SOTA models across various settings. Specifically, Table 1 presents the mean Dice scores and the corresponding inter-category performance deviations, providing a comprehensive assessment of performance distribution and its variability among the different anatomical categories. In the context of laparoscopic image segmentation, existing methods exhibit certain limitations. Notably, while some methods achieve results comparable to ours in learning new categories (8–9, 10–11) under some experimental conditions, 7–2 (2 steps) and 7–2–2 (3 steps), they significantly lag in retaining previously learned categories (1–7). To provide a detailed analysis of performance on each category, a comprehensive comparison of Dice scores for all categories after incremental learning of experiment 7–4 (2 steps) using the DeepLabV3+ framework is presented in Table 2.

**Fig. 4:**
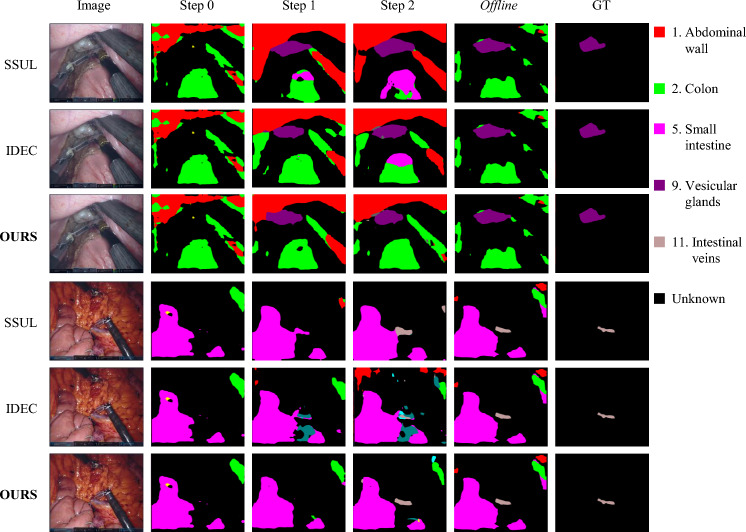
Experimental results from our 7–2–2 (3 steps) setup demonstrate the efficacy of our method in both retaining knowledge of previously learned categories and effectively learning new categories under incremental learning conditions. These findings highlight our network’s robust capability to manage the challenges associated with class-incremental learning in laparoscopic image segmentation

**Fig. 5:**
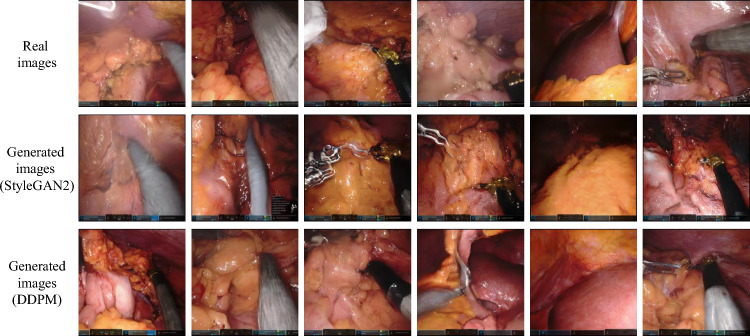
Comparison of real and generated laparoscopic images shows that the organ textures produced by the diffusion model appear remarkably realistic

We conducted ablation experiments to demonstrate the effectiveness of different components in our proposed method, as shown in Table 3. Due to the limited training data, methods based on DeepLabV3+ with fewer parameters yielded better results in our experiments. Both data generation (DG) and contrastive learning (CL) proved effective within the DeepLabv3+ and MedSAM frameworks. Specifically, using generated image data improves the results for previously learned categories. Contrastive learning particularly enhances performance for new categories. The potential reasons for this improvement might be the addition of feature alignment constraints to the network’s encoder and the enhanced ability to distinguish more effectively between new and old categories, which together contribute to better segmentation results for new classes.

Overall, our approach outperforms competing methods in most test scenarios. This underscores the efficacy of our incremental learning strategy in handling complex and difficult samples, illustrating its potential to enhance model performance in clinical image segmentation and related clinical applications.

### Qualitative evaluation

Figure 3 illustrates the segmentation results for all categories after incremental learning of experiment 7–4 (2 steps) using the DeepLabV3+ framework. Since the ground truth contains annotations for only one category, we include results from an offline method for comparative analysis to provide a benchmark of segmentation performance when all data are accessible at once. This comparison helps highlight the capabilities and limitations of our incremental learning approach compared to scenarios where all data are accessible. Our method not only achieves higher accuracy in segmenting annotated pixels, but also closely approximates offline segmentation performance for unannotated pixels. Additionally, it results in fewer scattered predictions. Figure 4 displays the predictions of different methods across various stages in the 7–2–2 (3 steps) experiment. Our method consistently provides more accurate predictions for newly introduced categories at each step and effectively retains the categories learned in previous steps.

**Table 4:** Quantitative comparison of images generated by StyleGAN2 and DDPM with real images

Method	IS $\uparrow$	FID $\downarrow$	Precision $\uparrow$	Recall $\uparrow$	Coverage $\uparrow$
StyleGAN2	3.01	108.13	0.076	0.450	0.058
DDPM	2.77	62.88	0.271	0.842	0.308

### Ablation studies about image generation models

The effectiveness of the diffusion model for image generation is further evaluated in Fig. 5, which presents a comparison between images generated by the DDPM and real images, demonstrating that DDPM excels in reproducing finer details. The ablation study, detailed in Table 4, contrasts two prominent image generation models, StyleGAN2 [31] and DDPM, across various performance metrics. While StyleGAN2 registers higher inception scores (IS), indicative of superior visual quality and diversity, the IS metric is derived from models trained primarily on non-medical images and may not fully capture the subtleties required for medical image generation. Conversely, DDPM surpasses StyleGAN2 in Fréchet Inception Distance (FID), suggesting that images generated by DDPM better preserve both the details and structure of real medical images, leading to more faithful visual representation. Additionally, DDPM demonstrates higher precision and recall, reflecting a more accurate reproduction and coverage of features found in the real image dataset. Further analysis includes using images generated by StyleGAN2 and DDPM within incremental learning with the DeepLabV3+ segmentation framework. The experimental results, detailed in Table 5, reveal that DDPM outperforms StyleGAN2 in CISS tasks.

**Table 5:** Dice scores performance when images generated by StyleGAN2 and DDPM are applied in experiment 7–2–2 (3 steps)

Method	1–7	8–9	10–11	1–11
KD	52.88	25.27	41.17	45.91
KD+DG (StyleGAN2)	59.86	28.57	43.87	51.26
KD+DG (DDPM)	63.86	29.32	42.13	53.63

## Conclusion

In this work, we tackle the challenge of class-incremental learning for identifying anatomical structures in laparoscopic images. By innovatively integrating a diffusion model for image generation, a distillation-based network framework, and contrastive learning techniques, we address issues arising from variations in appearance and binary annotation in laparoscopy. Our approach not only preserves knowledge of previously learned classes, but also effectively incorporates new classes. The effectiveness of our method is demonstrated through experimental results, establishing a noteworthy advancement in this emerging field. As a preliminary exploration, our future work will focus on refining the delineation of anatomical boundaries, further enhancing the applicability of class-incremental learning in laparoscopic image analysis.

## Supplementary Information

Below is the link to the electronic supplementary material.

Supplementary file 1 (pdf 52 KB)
