# Supplementary material for: Diffusion-driven distillation and contrastive learning for class-incremental semantic segmentation of laparoscopic images
Source: Int J Comput Assist Radiol Surg. 2025 Jun 14;20(7):1551–60. doi: 10.1007/s11548-025-03405-1 (PMC12226607; doi:10.1007/s11548-025-03405-1)
Supplement: Supplementary file 1 — (pdf 52 KB) [file 11548_2025_3405_MOESM1_ESM.pdf]

Diffusion-Driven Distillation and Contrastive Learning for Class-Incremental Semantic Segmentation of Laparoscopic Images: Supplementary Material

Table 1 Distribution of surgical cases across training, validation, and test sets following the officially recommended splits.

Dataset Split	Number of Surgeries	Surgery IDs
Training	21	1, 4, 5, 6, 8, 9, 10, 12,<br>15, 16, 17, 19, 22, 23, 24,<br>25, 27, 28, 29, 30, 31<br>(at least 12 surgeries per anatomical structure)
Validation	3	3, 21, 26<br>(3 surgeries per anatomical structure)
Test	8	2, 7, 11, 13, 14, 18, 20, 32<br>(5 surgeries per anatomical structure)

Table 2 Detailed breakdown of incremental learning scenarios showing the specific categories segmented in each step.

Scenario	Step	Categories Included
7-4 (2 steps)	Step 0	1. Abdominal wall, 2. Colon, 3. Liver, 4. Pancreas,<br>5. Small intestine, 6. Spleen, 7. Stomach
	Step 1	8. Ureter, 9. Vesicular glands, 10. Inferior mesenteric artery,<br>11. Intestinal veins
7-2 (2 steps)	Step 0	1. Abdominal wall, 2. Colon, 3. Liver, 4. Pancreas,<br>5. Small intestine, 6. Spleen, 7. Stomach
	Step 1	8. Ureter, 9. Vesicular glands
7-2-2 (3 steps)	Step 0	1. Abdominal wall, 2. Colon, 3. Liver, 4. Pancreas,<br>5. Small intestine, 6. Spleen, 7. Stomach
	Step 1	8. Ureter, 9. Vesicular glands
	Step 2	10. Inferior mesenteric artery, 11. Intestinal veins
